# Tissue-Tissue Interaction-Triggered Calcium Elevation Is Required for Cell Polarization during *Xenopus* Gastrulation

**DOI:** 10.1371/journal.pone.0008897

**Published:** 2010-02-02

**Authors:** Asako Shindo, Yusuke Hara, Takamasa S. Yamamoto, Masamichi Ohkura, Junichi Nakai, Naoto Ueno

**Affiliations:** 1 Department of Developmental Biology, National Institute for Basic Biology, Okazaki, Aichi, Japan; 2 Saitama University Brain Science Institute, Saitama, Saitama, Japan; 3 Department of Basic Biology, School of Life Science, The Graduate University of Advanced Studies (SOKENDAI), Okazaki, Aichi, Japan; The University of Hong Kong, China

## Abstract

The establishment of cell polarity is crucial for embryonic cells to acquire their proper morphologies and functions, because cell alignment and intracellular events are coordinated in tissues during embryogenesis according to the cell polarity. Although much is known about the molecules involved in cell polarization, the direct trigger of the process remains largely obscure. We previously demonstrated that the tissue boundary between the chordamesoderm and lateral mesoderm of *Xenopus laevis* is important for chordamesodermal cell polarity. Here, we examined the intracellular calcium dynamics during boundary formation between two different tissues. In a combination culture of nodal-induced chordamesodermal explants and a heterogeneous tissue, such as ectoderm or lateral mesoderm, the chordamesodermal cells near the boundary frequently displayed intracellular calcium elevation; this frequency was significantly less when homogeneous explants were used. Inhibition of the intracellular calcium elevation blocked cell polarization in the chordamesodermal explants. We also observed frequent calcium waves near the boundary of the dorsal marginal zone (DMZ) dissected from an early gastrula-stage embryo. Optical sectioning revealed that where heterogeneous explants touched, the chordamesodermal surface formed a wedge with the narrow end tucked under the heterogeneous explant. No such configuration was seen between homogeneous explants. When physical force was exerted against a chordamesodermal explant with a glass needle at an angle similar to that created in the explant, or migrating chordamesodermal cells crawled beneath a silicone block, intracellular calcium elevation was frequent and cell polarization was induced. Finally, we demonstrated that a purinergic receptor, which is implicated in mechano-sensing, is required for such frequent calcium elevation in chordamesoderm and for cell polarization. This study raises the possibility that tissue-tissue interaction generates mechanical forces through cell-cell contact that initiates coordinated cell polarization through a transient increase in intracellular calcium.

## Introduction

Convergent extension (CE), one of the most important cell movements in early vertebrate development, elongates the embryo along the anterior-posterior axis. During this process, chordamesodermal cells of the *Xenopus* gastrula become highly polarized, become spindle-shaped representing polarized intracellular events, and intercalate between each other mediolaterally, via active protrusions [1], [2], [3]. Although a signaling pathway initiated by secreted Wnt ligands, such as Wnt11, is essential for establishing the planar cell polarity (PCP) [4] that governs CE, little is known about the mechanism that initiates the coordinated polarity. In our previous study, we monitored the orientation of microtubule (MT) growth as an indicator of functional cell polarity, and demonstrated that cell-cell or tissue-tissue interaction plays a critical role in establishing cell polarity [5]. The most intriguing finding was that the polarity, the direction of MT elongation and cell alignment, becomes visible in chordamesodermal explants only when they are co-cultured in contact with a *Xenopus* embryonic tissue possessing different properties, *i.e.*, the lateral mesoderm or ectoderm. Furthermore, the polarity revealed by MT growth was evident only in the chordamesoderm, suggesting that mesodermal differentiation is prerequisite for the establishment of cell polarity. The importance of cell-cell or tissue-tissue interaction for coordinated cell behaviors such as cell migration and cell sorting has recently become a topic of intense interest [6], [7], [8]. The reports suggest that physical contact between two different tissues with distinct physical properties as regards cell adhesion and/or surface tension provides a cue for the initiation of cell polarity in cellular morphology and their alignment. These findings prompted us to investigate the very first event that occurs at the boundary of two distinct tissues, using contact cultures.

Previous reports demonstrated that a dorsally restricted calcium waves occur in the *Xenopus* gastrula embryo and that inhibition of the intracellular calcium release results in CE defects [9]. Intracellular calcium is thought to be a key factor regulating the direction of cell migration [10], the formation of cellular protrusions [11], cell-cell adhesion, and the modulation of gene expression [12], all of which take place during gastrulation. Moreover, intracellular calcium responds immediately after the cells are challenged by various extracellular stimuli, such as secreted growth factors [13], and perhaps as importantly, by physical contact [14]. Based on these findings, we investigated the relationship between intracellular calcium elevation and the formation of a tissue-tissue boundary between two distinct explants. Our results suggest a role for mechanical cues in the formation of chordamesodermal cell polarity during its transition to notochord.

## Results

### Conjugation of Distinct Tissues Causes Intracellular Calcium Elevation

To investigate intracellular calcium dynamics upon cell-cell or tissue-tissue contact, we used nodal-overexpressing animal caps, which were forced to differentiate into chordamesodermal cells [5]. G-CaMP 4.1, which is an improved form of a previously reported calcium indicator [15], was injected into the animal pole of two-cell-stage embryos along with various doses of nodal mRNA, and the calcium dynamics in the chordamesoderm were monitored by time-lapse imaging (Figure S1).

**Figure 1:**
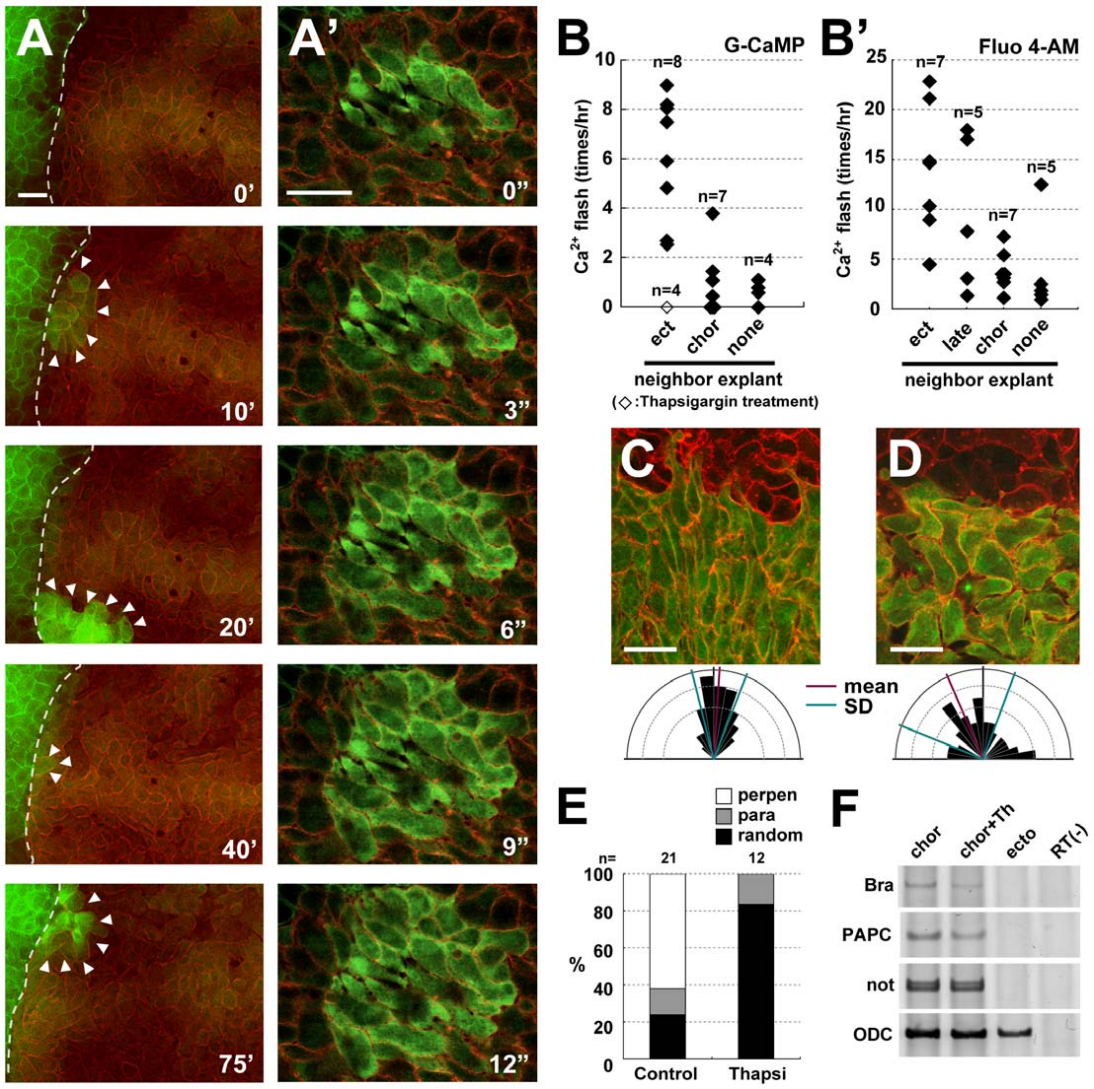
Increased intracellular calcium is induced in chordamesodermal cells by tissue attachment and is required for cell polarization. (A) Time-lapse analysis of the calcium dynamics in a heterogeneous conjugation assay taken by each 40 seconds. The right side of the explant (the cytoplasmic membranes were marked by RFP (red), co-injected with G-CaMP (green)) was chordamesoderm induced by the overexpression of nodal mRNA (150 pg), and the left side (the cytoplasmic membranes marked by GFP (green)) was ectoderm. The dotted line shows the boundary between the two explants, and the arrowheads indicate G-CaMP signals. (A′) The time-lapse images showing the calcium propagation indicated by G-CaMP in the chordamesoderm tissue taken by each 3 seconds. (B, B′) The frequency of calcium flashes in chordamesodermal tissues conjugated with heterogeneous neighboring explants, indicated by G-CaMP (B) or Fluo 4-AM (B′). Each diamonds show the average number of G-CaMP or Fluo-4 signals observed per hour in 10 cells at the boundary in each explants, and the open square in B shows the frequency under treatment with thapsigargin. (ect: ectoderm, chor: chordamesoderm, late: lateral mesoderm, none: no neighboring explant) (C) Perpendicular alignment of chordamesodermal cells in the conjugation assay with ectoderm. The upper part marked by membrane-RFP was ectoderm, and the lower part marked by GFP cytoplasm and membrane-RFP was chordamesoderm. The directions of the long axis of each cell were indicated by a rose diagram. (D) Abnormal cell alignment caused by thapsigargin treatment. The cells were aligned randomly compared with C. (E) Proportion of alignment types in relation to the border in the chordamesoderm shown in (C) and in the thapsigargin-treated sample shown in (D). (para: parallel, perpen: perpendicular) (F) RT-PCR analysis of mesodermal induction by the overexpression of nodal mRNA. Thapsigargin treatment did not affect the mesodermal induction. (bra: brachyury, Th: Thapshigargin) (bar: 50 µm).

The cells in the chordamesodermal tissues that were apposed to neighboring tissue, such as ectodermal or lateral mesodermal tissue, showed a high frequency of calcium flashes around the time of the first contact (Figure 1A and 1B, Movie S1). Live calcium imaging showed that the flash occurred when the cells of the two distinct explants made contact. It was confined to a single cell, or it propagated gradually through several surrounding cells (Figure 1A′). In contrast, intracellular calcium elevation was rarely observed when the chordamesoderm was cultured alone or as a homogeneous conjugation (i.e., another chordamesodermal explant) (Figure 1B, Movie S2). We observed similar calcium dynamics under the same conditions but using Fluo 4-AM, a chemical calcium indicator (Figure 1B′). The duration of the Fluo 4-AM signals was longer than that of G-CaMP. This might have resulted from their different calcium-dissociation profiles: G-CaMP has a higher Hill coefficient, despite its lower dissociation constant (Kd), than Fluo-4.

Because the calcium signals from G-CaMP were small and lasted only 10–20 seconds, the 40-second interval between the time-lapse recordings might not have detected some of them. Therefore, the absolute incidence of flashes was probably higher than we report here. Nevertheless, our data show that the calcium dynamics differed in heterogeneous and homogeneous conjugation cultures. Unlike Fluo 4-AM, which is added to cultures and must be taken up by the cells, G-CaMP was produced by essentially all cells of the explants, and its signal was clearer. Therefore, G-CaMP was better suited imaging calcium dynamics in *Xenopus* embryos. In our previous report, we tracked the movement of MTs with GFP-tagged EB3, a microtubule-binding protein, and showed that as similarly-induced chordamesodermal cells aligned perpendicular to an ectodermal or lateral mesodermal boundary, the MTs in the chordamesoderm elongated toward the boundary [5]. Our present results were consistent with these observations, and support the idea that the frequency of intracellular calcium elevation is increased by the formation of a boundary between two distinct tissues, such as between notochord and somitic mesoderm, which resembles the tissue alignment seen in the dorsal region of the normal embryo.

If the transient intracellular calcium elevation has a critical role in establishing cell polarity, calcium depletion would be expected to affect cell polarity formation. It was previously reported that CE is inhibited by the depletion of intracellular calcium stores [9]. To examine the role of calcium release in tissue-tissue interactions, we pre-treated chordamesodermal explants with the same inhibitor used previously, thapsigargin, which inhibits calcium uptake into the endoplasmic reticulum (ER) and eventually exhausts the intracellular calcium. We then observed the calcium response, the orientation of the elongated MTs, and the cell alignment in conjugated explants. The incidence of calcium flashes near the boundary of heterogeneous conjugates was significantly decreased by the thapsigargin treatment (Figure 1B open square). In addition, in the inhibitor-treated explants, the cells aligned randomly relative to the boundary (Figure 1D, E) rather than perpendicularly (Figure 1C, E). EB3-GFP showed the direction of MT elongation was more radial (Movie S3) than in the explants without inhibitor, whereas the induction of the chordamesoderm was unaffected (Figure 1F). These results strongly suggest that intracellularly stored calcium is required for the transient calcium elevation that contributes to the polarization of chordamesodermal cells.

### Collective Interaction of Heterogeneous Cells Is Essential for the Calcium Elevation

**Figure 2:**
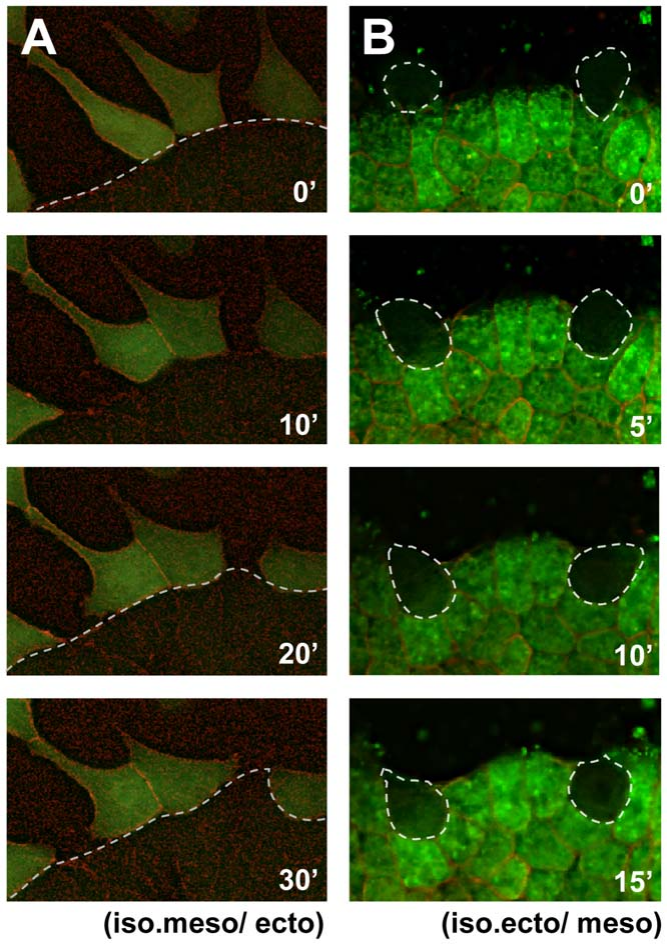
The cell mass is required for the intracellular calcium increase in chordamesodermal cells. (A) Single frames picked from the time-lapse analysis of calcium dynamics in isolated single chordamesodermal cells (G-CaMP (green cytoplasm) and membrane RFP (red)) upon their attachment to ectodermal tissue (below the dotted line, membrane RFP) taken by each 40 seconds. (B) Calcium dynamics of the chordamesodermal tissue (Fluo 4-AM (green cytoplasm) and membrane RFP (red)) when isolated single ectodermal cells (surrounded by a dotted line) were attaching to it. The same experiments were performed using G-CaMP. They did not show the active signals in either (A) or (B). (iso.meso: isolated chordamesoderm, ecto: ectoderm, iso.ecto: isolated ectoderm, meso: chordamesoderm).

When two explants first touched, calcium flashes were often activated in single cells (Movie S1). It was therefore possible that the calcium elevation was triggered by molecular interactions between cell-surface components, such as ligands and receptors, of individual cells from the heterogeneous tissues. Therefore, we next examined whether isolated cells from a chordamesoderm-fated explant could trigger calcium elevation upon contact with a heterogeneous tissue. The single chordamesodermal cells did not change their calcium level upon contacting the heterogeneous tissue (Figure 2A, Movie S4), indicating that they needed to be part of a cell mass to elevate their intracellular calcium. The attaching isolated chordamesodermal cells were observed for 1.5 to 2 hours (n = 26), and calcium increase was detected only in one chordamesodermal cell. We also tested whether single cells of the ectoderm-fated explant could trigger a calcium flash in chordamesodermal tissue. Interestingly, the single cells isolated from ectodermal explants also failed to cause regional intracellular calcium flashes in the neighboring chordamesoderm upon their direct contact (Figure 2B, Movie S5). The attaching isolated ectodermal cells were observed (n = 27), and only one ectodermal cell caused calcium increase in the chordamesodermal tissue. Taken together, these findings indicated that both the stimulating and responding cells must be in cell masses to increase the intracellular calcium in the chordamesodermal cells, and suggest that their physical contact, rather than the simple protein-protein interaction of cell-surface components, is essential for the calcium response.

### Contact Geometry of Tissues May Be Key for Polarity Formation

**Figure 3:**
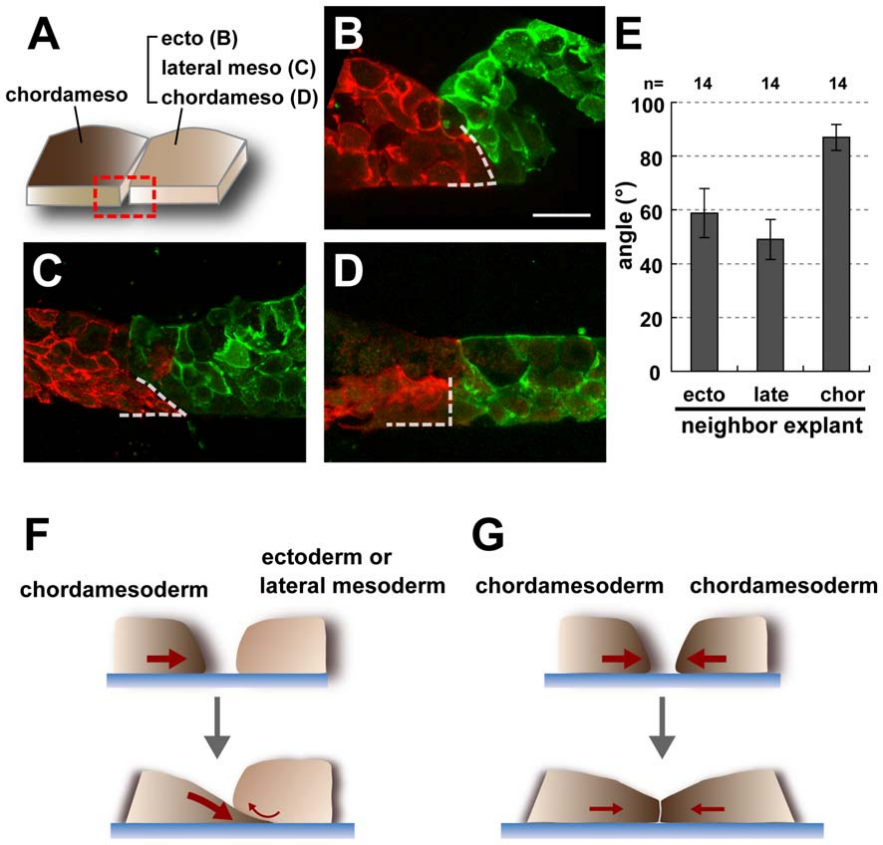
Geometry of the vertical section of the explants in the conjugation assay. (A) Diagram showing the area observed by section. The red box indicates the observed area, which is the vertical plane of the boundary between the chordamesoderm and the contiguous tissue. (B, C, D) Vertical sections of the chordamesoderm (red) and its neighboring tissue (green). The chordamesodermal tissue had a wedge-like shape (dotted lines)when conjugated with ectoderm (B) and lateral mesoderm (C), but the edge of the tissue remained essentially vertical in homogeneous conjugation assays (D). (E) Graph showing the average angle of chordamesodermal tissues conjugated with neighboring tissues. (n = 14 in each group, ecto: ectoderm, late: lateral mesoderm, chor: chordamesoderm) (F, G) Model for the creation of the boundary geometry. The chordamesodermal tissue crawled under the ectoderm or lateral mesodermal tissues because of its strong adhesion to the fibronectin, creating a wedge-like shape, and attachment of the apical side of the chordamesodermal cells to the neighboring explant (F). On the other hand, the orthogonal-like shape was formed because of the cells' equal adhesion to the fibronectin when chordamesoderm was conjugated to itself (G).

Next, we carefully investigated the morphological differences and compared the geometry of the contacting surfaces of homogenous chordamesoderm-chordamesoderm conjugates and heterogeneous conjugates between chordamesoderm and ectoderm (naïve animal cap) or lateral mesoderm (Figure 3A). By optical sectioning across the contact surface, we found that the geometric arrangement of the contacting tissues was unique: the chordamesodermal cells were organized so that the part of the explant in contact with the heterogeneous tissue formed a wedge, or beveled edge, that was tucked beneath the apposed portion of the heterogeneous tissue (Figure 3B, C, E). This configuration might have been caused by the stronger adherence of the chordamesodermal cells to the fibronectin and their higher motility on this substrate, compared with the other cell type. In contrast, the contact surface between the homogeneous chordamesodermal tissues was nearly vertical to the substrate layer (Figure 3D, E).

We also observed that the chordamesodermal cells underlying the ectoderm or lateral mesoderm were often highly protrusive, extending lamellipodia at their leading edges (data not shown). Lamellipodia are thought to form focal adhesions on the fibronectin-coated bottom of the culture dish. In chordamesodermal cells with little protrusive activity, the intracellular calcium was not elevated with high frequency (data not shown). These observations led us to speculate that the chordamesodermal cells near the boundary experience mechanical stimuli such as stretch stress generated by their own motility during migration leading to the deformation of the cells. (Figure 3F, G).

### Intracellular Calcium Flash Is Triggered by Mechanical Stress

**Figure 4:**
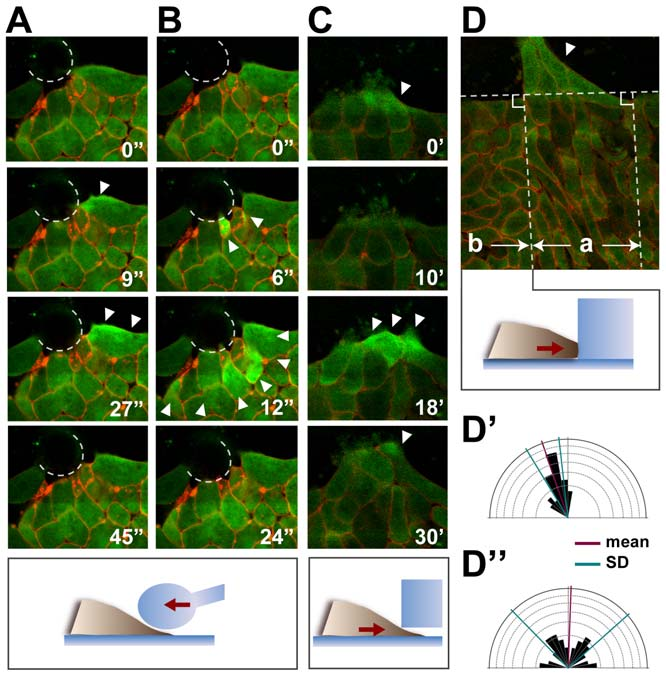
Mechanical stimuli change the intracellular calcium dynamics and cell polarity. (A, B) Increase in the intracellular calcium in the chordamesodermal tissue by glass needle pushing. The dotted lines show the edge of the glass needle. Before the pushing of the glass needle (0 sec), the calcium did not show any activated signals. Just after the pushing of the needle (9 sec in A, 6 sec in B), the cells responded to the stimulus by an increase in intracellular calcium (arrowheads). The calcium propagation was either restricted to a single cell (A), or to several cells (B), and disappeared within a minute. (C) Increase in intracellular calcium (arrowheads) in the chordamesodermal tissue by cells crawling underneath a block of silicone. (D) Cell alignment after silicone block attachment. One hour after the attachment in (C), the cells behind the crawling cells (arrowhead) aligned perpendicular to the silicone block (area indicated by a). The horizontal dotted line indicates the edge of the silicone block. (D′, D″) Rose diagrams showing the distribution of the cells' angles in D. The cells behind the cells that received the stimulus (a in D) aligned almost perpendicular to the edge of the silicone block (D′), but cells that did not receive the stimulus (b in D), did not (D″).

It is known that intracellular calcium can be elevated by various mechanical stimuli in cultured cells [14], [16], [17]. To assess whether the *Xenopus* chordamesodermal cells also respond to mechanical stimuli, we applied a mechanical force by pushing the chordamesodermal tissue with a glass needle with a heat-blunted tip. We observed that the intracellular calcium in a single or a few chordamesodermal cells was transiently increased upon their initial contact with the tip (Figure 4A, B). The flash was confined to a single cell (Figure 4A, Movie S6) or propagated to surrounding cells as a wave, and disappeared within one minute (Figure 4B, Movie S7). Interestingly, in some cases, the cells became elongated parallel to the direction of the mechanical force after a few hours of physical contact (data not shown), suggesting that the propagation of the calcium wave was sufficient to confer cell polarity on the chordamesoderm. However, such alignment was rarely observed after a simple needle push, probably because the force conferred by the needle did not recapitulate the endogenous one. A likely scenario based on this observation is that in vivo, the chordamesodermal cells respond to mechanical stresses caused by their own motility when they form adhesive contacts with ectodermal or lateral mesodermal tissues.

We next tested whether a block of silicone could affect the intracellular calcium dynamics in chordamesodermal cells that attached to it spontaneously, that is, via their own motility and without external forces. When the chordamesodermal cells attached to the silicone wall surface, clear calcium flashes were infrequent. Interestingly, however, an obvious elevation in intracellular calcium was observed when the cells crawled into a small gap between the silicone block and the bottom of the glass dish (Figure 4C, Movie S8). After a few hours, the cells aligned perpendicular to the silicone block behind the region where 2–4 layers of tip cells were located underneath the silicone in 66.7% of explants (n = 6, Figure 4D, D′). The other areas of the explant did not show such coordinated alignment (Figure 4D, D″). Similar results were obtained in other situations; a silicone block with a minute furrow at its edge caused frequent calcium flashes and coordinated alignment in that area (n = 7, 71.5% of the explants, Figure S2, Movie S9). These results strongly suggested that the sliding of the cells underneath the silicone block provided a similar mechanical stimulus to the one seen in the heterogeneous tissue conjugation, which has a key role in increasing intracellular calcium and initiating cell polarization.

### Dorsal Mesodermal Cells Show Transient Intracellular Calcium Flashes Near the Notochord-Somite Boundary

**Figure 5:**
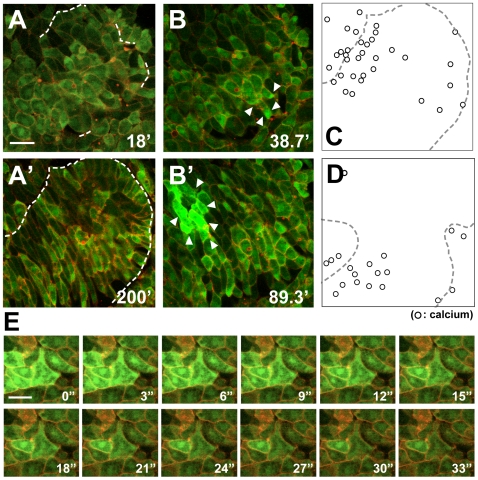
Calcium signals in the notochord-somite boundary area. (A, A′) Single frame picked from the time-lapse analysis of CE in the DMZ taken by each 40 seconds (Movie S10). The cells were rounded at st. 11.5 – 12, and the boundary was partially visible (A). The cells elongated and intercalated with each other to form the notochord after about 3–4 hours, and the boundary was clearly formed (A′). (B, B′) Single frames picked from the same movie as (A) and (A′). The calcium signals near the boundary indicated by an arrowhead. (C) Locus of the cells in the last frame that showed calcium increase during (A) to (A′) were indicated by dots. The dotted line indicates the boundary at stage (A′). (D) Another explants showing the same tendency as (C). (E) The sequential shots from time-lapse movie of calcium signals in DMZ taken by each 3 seconds. (bar: 50 µm).

We next sought to identify the locus of calcium elevation during convergent extension in the isolated DMZ at single-cell resolution. To examine the spatio-temporal dynamics of calcium release, time-lapse recordings were performed from early in the formation of the notochord-somite boundary (Figure 5A) to the late stages of boundary formation (Figure 5A′). At hours 3–4, some cells showed large calcium waves and others showed smaller ones (Figure 5B, B′, Movie S10), as previously described [9]. A plot of the cells showing the calcium signals revealed that the areas with frequent calcium elevations were well overlapped with the region where the notochord boundary was established (n = 7, 71% of calcium flashes, Figure 5C, D), and that some cells showed smaller flashes when they attached to the newly formed boundary. Conversely, it was rare that cells ending up in areas far from the boundary had such calcium increases. A calcium flash had a duration ranging from about 10 to 90 sec as the propagation (Figure 5E), and usually occurred only once in an individual cell of earlier stages, and large calcium waves (lasting over 2 minutes in area over 10 cells) continued in later stages.

It was previously shown that the calcium waves and flashes occurring in the DMZ are required for normal convergent extension [9]. In addition, the intracellular calcium released from the ER is known to be critical for normal gastrulation. Taking these findings together with ours, we propose that the calcium flashes coincide with boundary formation and contribute to the establishment of cell polarity during *Xenopus* gastrulation.

### Purinergic Receptor P2Y11 Is Essential for Convergent Extension

**Figure 6:**
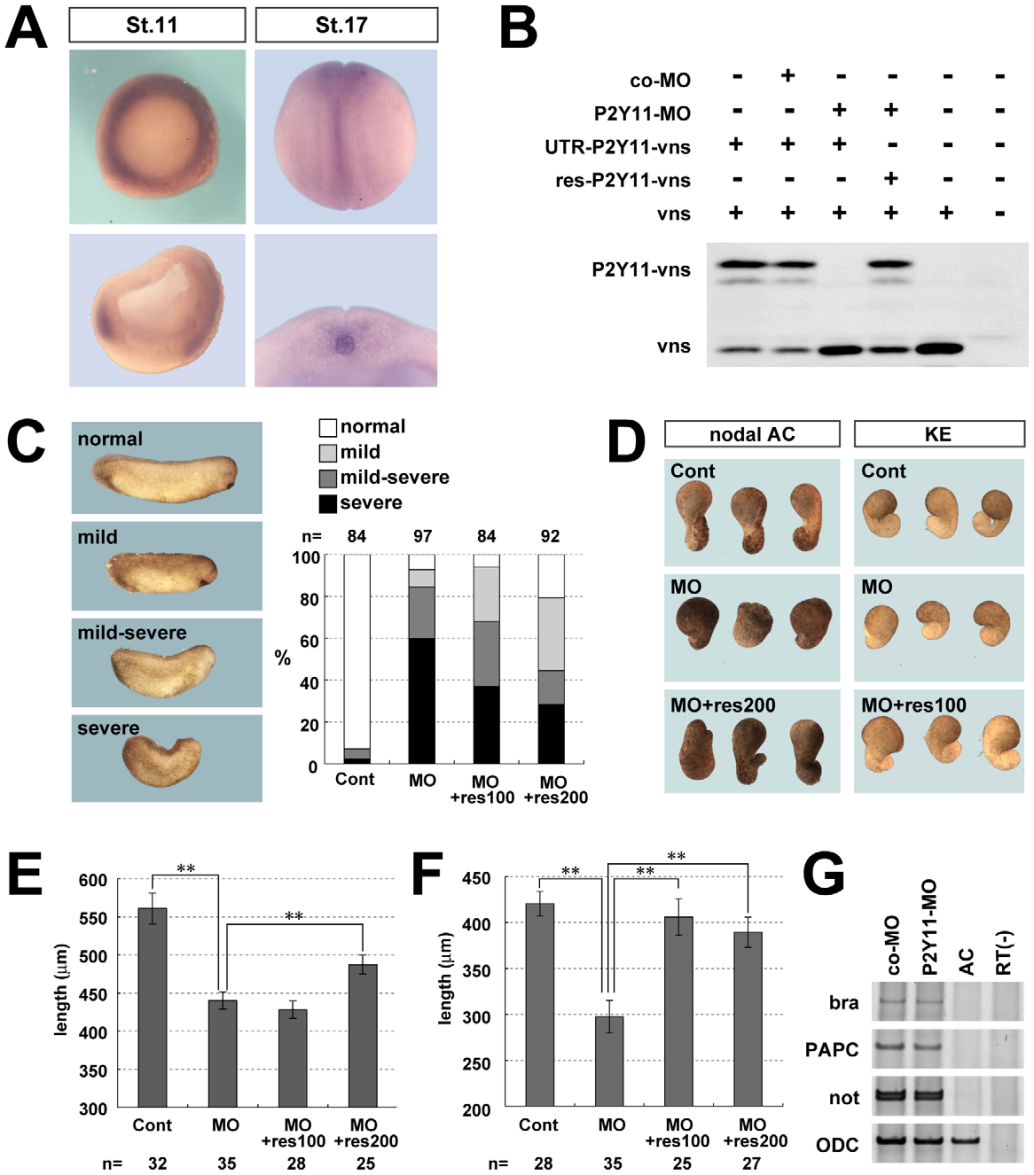
P2Y11 was expressed in the notochord, and was required for normal CE. (A) Expression pattern of P2Y11, from the dorsal view (upper), and the cross section of the whole embryo at stage 11 and of the dorsal region at stage 17 (lower). (B) In vitro transcription/translation system showing the effect of P2Y11-MO on the transcription of P2Y11 mRNA. P2Y11-MO specifically inhibited the transcription of UTR-including P2Y11 mRNA (UTR-P2Y11-vns), constructed to generate a fusion protein with eYFP (vns). The effect of P2Y11-MO was restored by coexpression of a rescue construct (res-P2Y11-vns). P2Y11 protein was detected by western blotting with an anti-GFP antibody. Vns protein was detected as a loading control. (C) The proportion of phenotypes in control or P2Y11 morpholino (30 ng) -injected embryos at stage 28. Full length of P2Y11 mRNA lacking MO-targeting site (res- P2Y11 mRNA) rescued the phenotype with dose dependency. (D) Inhibition of nodal-expressing animal cap and Keller Explant elongation by the P2Y11-morpholino. The inhibition was rescued by res- P2Y11 mRNA overexpression. (E) The average length (µm) of nodal-expressing animal caps. The elongation of animal cap was attenuated by P2Y11-morpholino and rescued by res- P2Y11 mRNA overexpression. (** p<0.01) (F) The average of the neck length (µm) of Keller Explant with control or P2Y11 morpholino. The P2Y11-morpholino-injected explants were shorter and thicker than control morpholino-injected explants, and res- P2Y11 mRNA rescued its inhibition. (** p<0.01) (H) RT-PCR analysis of the nodal-overexpressing animal cap.

Given that mechanical stress is the key to cell polarization and proper morphogenesis of the notochord, embryonic cells should respond through mechano-sensing receptors. So far, it has been shown that cells responding to a mechanical stimulus release nucleotides (ATP or UTP), which trigger the increase of intracellular calcium; members of the purinergic receptor Y (P2Y) family function as the receptor for ATP in this process [18], [19], [20]. Based on this knowledge, we used in situ hybridization to investigate whether the notochord expressed a receptor for ATP. Indeed, the ATP receptor P2Y11 [21], which was reported to be induced by activin overexpression [22], was expressed in the dorsal mesoderm in early stages and specifically in the notochord in later stages (Figure 6A). To examine the contribution of P2Y11 to CE, we used a P2Y11-specific morpholino antisense oligonucleotide to deplete P2Y11 (Figure 6B).

Embryos in which P2Y11 translation was inhibited showed gastrulation defects, including a short and curved trunk (Figure 6C), and spina bifida by a high dose of morpholino (42 ng). The nodal-overexpressing animal caps did not elongate as the control caps by morpholino (Figure 6D, E), and the elongation of Keller explants was attenuated showing thick necks of mesodermal tissue (Figure 6D, F), despite the normal mesodermal induction (Figure 6G). All of these phenotypes were partially rescued by the co-expression of P2Y11 that lacks the target site of the morpholino (Figure 6D, E, F), suggesting that P2Y11 contributes to the proper CE during *Xenopus* gastrulation.

### Purinergic Receptor P2Y11 Is Essential for the Intracellular Calcium Elevation and Cell Polarization

**Figure 7:**
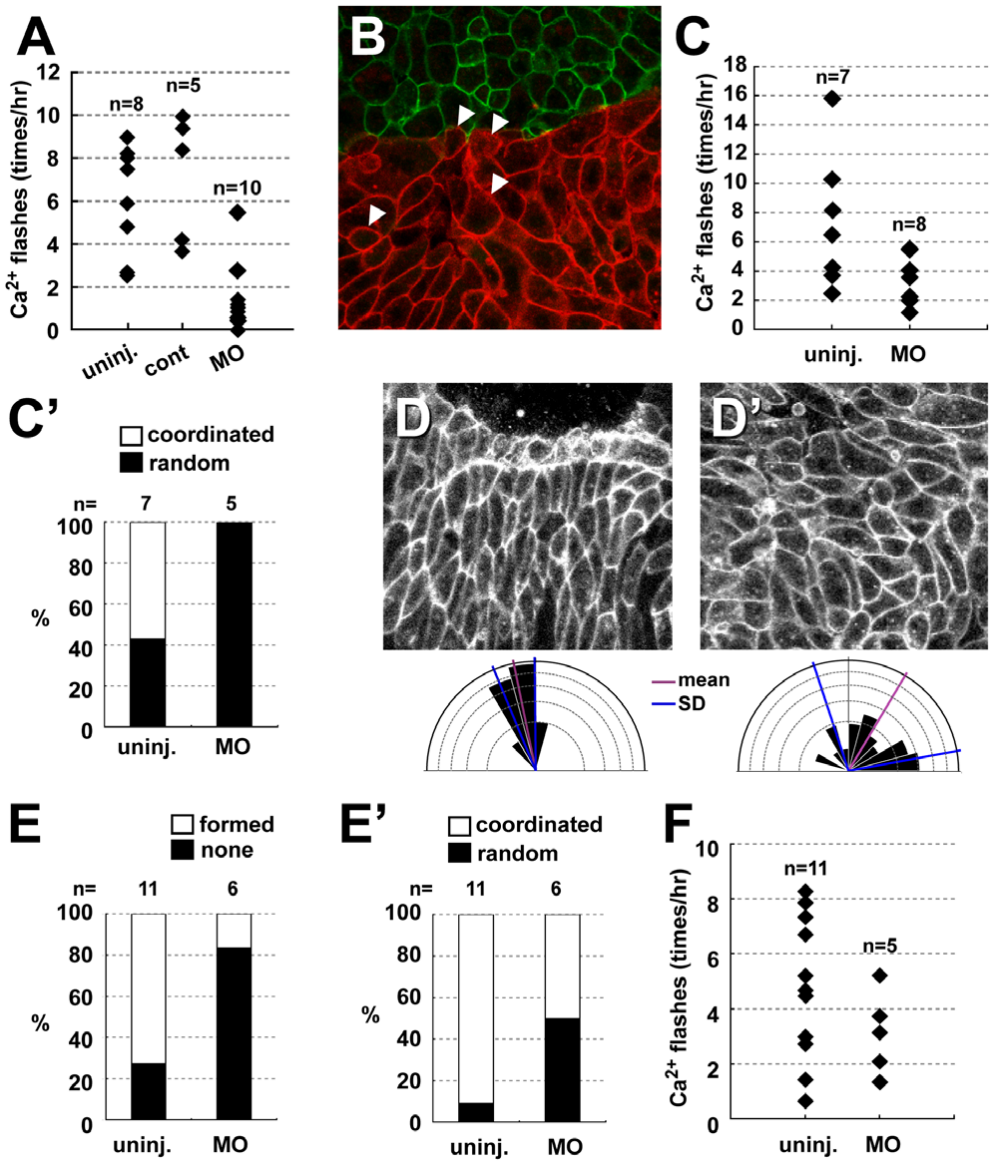
P2Y11 is required for frequent calcium elevation and coordinated cell polarization. (A) The effect of P2Y11-MO on the frequency of calcium flashes in chordamesodermal tissues near the boundary with ectoderm explants. (uninj.: morpholino-uninjected, cont: control morpholino, MO: morpholino) (B) The cell alignment in the P2Y11-MO-injected chordamesodermal tissue (RFP-membrane (red)) near the boundary with ectoderm (GFP-membrane (green)). The several rounded cells were contained (arrowheads) and the perpendicular alignment was relatively disturbed. (C) The effect of P2Y11-MO on the frequency of calcium flashes in the chordamesodermal tissues at the area of crawling cells under the furrow of silicone block. (C′) The ratio of cell alignment in (C), and coordinated cell alignment was not established in the MO-injected explants under the silicone furrow. (D, D′) cell alignment after CE in wild type DMZ (D), and morpholino-injected DMZ (D′). The cells showed coordinated alignment with proper polarity in D while their polarity was disrupted in D′. The lower rose diagrams show the angles of long axis of each cells in (D) and (D′). (E) The ratio of coordinated cell alignment in wild type DMZ and P2Y11-MO-injected DMZ. In the morpholino-injected DMZ showed random cell alignment compared with wild type. (E′) The ratio of boundary formation in wild type DMZ and P2Y11-MO-injected DMZ. Most of the morpholino-injected explants did not form clear boundary. (F) The number of calcium flashes in wild type DMZ and P2Y11-MO-injected DMZ.

Most importantly, the morpholino-injected chordamesodermal explants, when conjugated with heterogeneous explants, showed less frequent calcium flashes (Figure 7A) compared to the uninjected or control-morpholino injected groups. The alignments were relatively randomized containing rounded cells in the chordamesodermal tissue near the boundary when they conjugated with heterogeneous tissue (Figure 7B). We also found that the frequency of calcium elevation caused by touching and crawling the furrow of silicone block was attenuated by depletion of P2Y11 function (Figure 7C), and coordinated alignment in the crawling area was disturbed (Figure 7C′). Finally, we observed that the normal boundary formation (Figures 7D, D′, E) and coordinated cell alignment (Figures 7D, D′, E′) was not clear in the P2Y11-depleted DMZ, and the frequency of calcium elevation during CE was relatively decreased (Figure 7F).

These results suggest that P2Y11 may contribute to mechano-transduction indirectly via nucleotide and constant calcium increase at least partly in proper CE during *Xenopus* gastrulation. These results support our hypothesis that the mechanism of polarization and CE that gives rise to the notochord is initiated by mechanical stimuli transduced by intracellular calcium dynamics.

## Discussion

Our present results, together with those of our previous report, suggest that the boundary formed between two contiguous tissues, namely the chordamesoderm and lateral mesoderm or chordamesoderm and ectoderm, creates a cue for cell polarization. In our previous report, we showed that contact of two tissues, which received different levels of nodal and differentiate into two distinct cell fates such as ectoderm and mesoderm, is important to generate the microtubule polarity and cell alignment. In this study, we focused on the intracellular calcium dynamics near the tissue boundary, which were necessary for cell polarization of the chordamesoderm and CE. The frequency of calcium elevation in the chordamesoderm was dependent on the nature of the neighboring tissue, consistent with our previous observation that the cell alignment and MT elongation establish their polarity according to the boundary with a neighboring tissue. Because one of the earliest cellular responses to an external signal is a calcium event, this could be the earliest response in the cascade that leads to cell polarization.

A major question is what feature of the boundary acts as the initiator of cell polarization. One possibility is that secreted factors from the heterogeneous neighbors contribute to cell polarization in the chordamesoderm. However, because single chordamesodermal cells did not show calcium elevation when contacting heterogeneous tissue, such secreted factors are unlikely to be the cue for calcium alterations, assuming that the isolation process did not greatly affect the physiological function of the cells. A second possibility is that the cell-cell interaction via cell-surface components triggers the cell behavior. This is also unlikely, because single ectodermal cells failed to elevate the intracellular calcium in chordamesoderm, and dissociated chordamesodermal cells failed to elevate it upon contact with ectoderm. A third possibility is that a difference in the physical properties of the contiguous tissues is essential for the initiation of cell polarity.

We showed that the cells in *Xenopus* explants could respond to a mechanical stimulus. The similarity of the calcium elevation induced by a mechanical force and by the culture of contiguous tissues led us to speculate that the neighboring tissue provides a mechanical stimulus to the chordamesoderm. In support of this idea, both the mechanical stimulus and the tissue-tissue interaction induced a similar calcium-response profile, consisting of small calcium flashes as well as larger waves, both of which were suppressed by depletion of the calcium store in the ER. Finally, we demonstrated that the calcium dynamics in the DMZ during notochord formation showed a similar type of calcium wave near the boundary. The spike in intracellular calcium upon mechanical stimulus from neighboring tissues may inform the cells about the direction of the boundary, information that is required for the later polarization of MT growth [5]. The mechanical stimulus also could provide topological information about the mediolateral axis in vivo.

Given that a mechanical stimulus is received by the chordamesodermal cells in vivo, we can speculate about how such a force is generated. The chordamesodermal cells move actively, but their strong adherence to the substrate causes them to crawl beneath the neighboring tissue. The contact phase appears to be essential, because a silicone block increased the intracellular calcium only when the chordamesodermal cells happened to burrow under it; a minute furrow in the block could also confer calcium elevation and cell polarization. These observations suggest that the interaction of the chordamesodermal cells with a different tissue changes their mechanical environment such as stretching of cytoplasmic membrane, which triggers the increase in intracellular calcium.

Cells of different embryonic lineages have differing levels of surface tension and stiffness, depending on the polymerization of their cortical actin [7], [23], suggesting that when cells differentiate into chordamesoderm and lateral mesoderm, in response to nodal signaling, the two tissues exhibit distinct levels of cell cortex tension. In addition, the elasticity of the substrate can affect cell differentiation [24] and cell behaviors, such as cell motility [25], [26], indicating that the microenvironment provides a cues that that direct cells' response and behavior. These observations, taken together with our data, suggest that mechanical stress is exerted on the cytoplasmic membrane when two tissues of different stiffness or surface tension contact each other with a specific geometry, as seen in the conjugation of chordamesoderm and a heterogeneous tissue (ectoderm or lateral mesoderm). Furthermore, such topology could also be attained in vivo, because the embryo has fibronectin fiber in the blastocoel roof, where the chordamesoderm crawls during gastrulation. Therefore, based on our present results, we hypothesize that a mechanical interaction generated at the interface of two tissues, namely the notochord-somite boundary, causes the membrane to stretch in a specific region and initiate cell polarization, and that this interaction is indispensable for the initiation of cell polarization.

The remaining question we addressed was whether receptors for mechanical stimuli were expressed in vivo in a spatio-temporal pattern that was consistent with our hypothesis. Importantly, we showed that a P2Y receptor, P2Y11, was expressed in the notochord and was required for normal convergent extension, coordinated cell polarization, and full elevation of the intracellular calcium in chordamesodermal cells in heterogeneous combinations. Current evidence indicates that P2Y1 and P2Y2 are required for intracellular calcium elevation in response to mechanical stimuli in various cell types [20], [27]. In these cases, nucleotides (ligands of P2Y) are released by the mechanical stimulus and bind the P2Y receptors, leading to an elevation in intracellular calcium via an IP_3_-dependent process [21]. In *Xenopus*, P2Y11 is expressed in the dorsal region from the early gastrulation stage, and activates calcium and cAMP signaling [22]. Therefore, it is possible that P2Y11 also activates the calcium signaling via nucleotide release during embryogenesis, even though it shares only weak homology with P2Y1 and P2Y2. Indeed, we showed that the depletion of P2Y11 attenuated the frequency of the transient increase in intracellular Ca^2+^ in the chordamesoderm of conjugate culture, and by crawling under the silicone block. Unfortunately, however, we were unable to demonstrate in this study that the depletion of P2Y11 blocks the intracellular Ca^2+^ elevation in the chordamesoderm by applying force with a needle. This may suggest that P2Y11 is not the direct sensor to detect the mechanical stimulus, or the force applied with the needle was rather strong compared to the force generated in vivo. For better understanding of the exact contribution of other P2Y family receptors and mechano-sensing receptors to the Ca^2+^ elevation, precise control of the applied force at magnitudes comparable to the in vivo situation is necessary. Although several issues remain to be resolved, the possibility that tissue-tissue interaction during embryogenesis causes mechanical stress opens a new avenue for studying the cellular mechanisms of organ morphogenesis.

## Materials and Methods

### Embryo Manipulations

*Xenopus* eggs were collected as described [28], and the embryos were staged according to Nieuwkoop and Faber [29]. The animal caps or Keller explants were excised at stage 9 or 10.5 respectively, then placed in 0.1% bovine serum albumin (BSA)/1x Steinberg's solution and mounted on glass dishes coated with fibronectin (FN) (0.5 mg/ml, F1141; Sigma-Aldrich) for observation by confocal (Carl Zeiss LSM510) or fluorescence (Olympus IX81) microscopy.

### Ethics Statement

All the treatments of animals in this research followed the guideline of NIBB and were approved by the committee.

### Antisense Morpholino Oligonucleotides

The antisense morpholino oligonucleotides (MOs) were obtained from Gene Tools (Plilomath, OR). The MO sequence was follows: P2Y11-MO 5′-gcagacggaggaagccatttattct-3′.

### Plasmid Construction and mRNA Preparation for Microinjection

GFP-fused EB3 was a gift from Dr. Akhmanova. The construct was digested with the appropriate restriction enzyme and inserted into the pCS2p+ plasmid. Venus-fused P2Y11 including the 5′UTR (−62 bp to 234 bp from ATG) was constructed by PCR (primer: upstream, cacgcgtccggcgggaaccag, downstream, catggtatagtaggccaccag, from AM040941), and inserted into the pCS2^+^-Venus plasmid. Venus-fused P2Y11 or full length P2Y11 including 6-mis 5′UTR (agattagatggcatcgtctgtgtgc) were also inserted into pCS2^+^ plasmid. The plasmid was linearized with Not I for transcription. Capped mRNAs were synthesized using the mMESSAGE mMACHINE SP6 kit (Ambion), and purified on a NICK column (Pharmacia, Uppsala, Sweden) before being injected into four-cell-stage embryos.

### Reverse Transcription-Polymerase Chain Reaction (RT-PCR)

RT-PCR was carried out as reported [28]. The PCR primer sequences used for the analysis of Xnot, Xpapc, and ODC, an internal input control, were as previously described [30], [31] [http://www.hhmi.ucla.edu/derobertis/]. The expression of each molecular marker was detected by PCR using the following specific primers: Xbra, upstream, ggatcatcttctcagcgctgtgga, and downstream, gttgtcggctgccacaaagtcca. For the analysis, 7 animal cap explants injected with nodal mRNA were detached at stage 9, and assayed when the sibling embryos reached stage 15.

### Calcium Imaging in the Conjugation Assay

The conjugation assay was performed as described [5]. Briefly, 150 pg of nodal-expressing animal cap, which was co-injected with G-CaMP 4.1 and membrane-RFP, was cut out at stage 9 as the chordamesoderm tissue, mounted onto a fibronectin-coated glass dish in Steinberg's solution, and a normal animal cap or 30–40 pg of nodal- expressing animal cap was mounted next to it as the ectoderm or lateral mesoderm, with a narrow gap between them to observe the timing of their attachment by confocal (Carl Zeiss LSM510) or fluorescence (Olympus IX81) microscopy (Figure S1). To apply Fluo-4 AM (Invitrogen) to visualize the calcium dynamics, the nodal-expressing animal cap was dipped into Steinberg's solution mixed with Fluo-4 AM (presumptive concentration: 1 µg/100 µl) and cultured for 3–4 hours with the detergent, cremophor. After washing out the Fluo-4, the adjacent tissues were placed beside the nodal-expressing animal cap, and observed.

#### Calcium imaging in conjugation assays with single cells

The nodal-expressing animal cap or normal animal cap was dipped into the Ca^2+^-, Mg ^2+^-free medium for 30 minutes to dissociate the cells. The medium was then changed to normal Steinberg's solution, and the substrate, which was the neighboring tissue, such as normal animal cap or nodal-expressing animal cap, was placed beside the cells. The culture was left at rest until the cells showed attachment to the substrate.

### Mechanical Stimulus Analysis

The glass needle was attached to a three-dimensional manipulator and pushed gently toward the G-CaMP-expressing explants. The tip of the needle was rounded by a flame to avoid wounding the cell. The silicone blocks were made of Sylgard 184 Silicone Encapsulant (Ellsworth). After mixing the base and curing agents and pouring the solution into a culture dish, the air was removed in vacuo, and the dish was left stationary at 50°C to allow the silicone to solidify. The piece of silicone was cut out, placed beside the nodal-expressing animal caps, and held down on the bottom of the dish by a cover glass.

### Calcium Imaging in Convergent Extension

Live-color imaging of *Xenopus* embryos was carried out as described [32], with minor modifications. Briefly, 1000 pg of G-CaMP and 250 pg of membrane-RFP, used as a marker, were injected at the dorsal side of four-cell-stage embryos. Keller explants were isolated from stage 10.5 embryos and cultured in 0.1% BSA/1× Steinberg's solution in a glass-bottomed dish coated with fibronectin, at 13°C for 6–10 hrs. The observation was initiated before the cells became spindle shaped, and was performed with a laser-scanning confocal microscope (Carl Zeiss LSMf510). Time-lapse images was recorded every 40 sec for 5–6 hours.

### *In Situ* Hybridization

Whole-mount in situ hybridization was performed with digoxigenin (DIG)-labeled probes, as described in [33]. Hybridization was detected with an alkaline phosphatase-coupled anti-DIG antibody and visualized using BM purple (Roche Molecular Biochemicals). The antisense in situ probe against P2Y11 was generated from a pBlueScript SK(−) plasmid encoding XL421a16, isolated from XDB3 of the NIBB/NIG *Xenopus* EST database (http://xenopus.nibb.ac.jp/). The linearizing enzyme for each construct was BamHl.

## Supporting Information

Figure S1 Experimental methods (time-scale). See method “Calcium imaging in the conjugation assay”
(0.42 MB TIF)

Figure S2 Cell alignment was altered where the apical side of the cells was underneath a silicone block. (A) The edge of a silicone block was scratched with a needle to create a furrow of the size indicated, and chordamesodermal tissue was placed beside it. (B) The cell alignment underneath the silicone furrow was coordinated (a) compared with the other area (b) (black area is the silicone block). (C) The angle of the long axis of the cells in area (B -a) was measured and is shown as a rose diagram. The underlying cells in the furrow tended to elongate and showed coordinated alignment. (D) The rose diagram showing the angle of the long axis of the cells in area (B - b). They did not show coordinated alignment. (E) The calcium flashes were observed near the furrow with high frequency (Movie S9). The dots show the sites of calcium flashes in the chordamesodermal tissue for 4 hours while cells were crawling under the silicone block (black area is the silicone block), and the number of them is indicated in the graph.
(1.30 MB TIF)

Movie S1 Calcium dynamics of chordamesodermal cells in heterogeneous conjugation. The right side of the explant (the cytoplasmic membranes are marked by red) was chordamesoderm induced by the overexpression of nodal mRNA coinjected with G-CaMP, and the left side (marked by green) was ectoderm. The cells in the chordamesodermal tissues showed a high frequency of calcium flashes around the time of the first contact.
(6.57 MB AVI)

Movie S2 Calcium dynamics of chordamesodermal cells in homogeneous conjugation. The right side of the explant (the cytoplasmic membranes are marked by red) was chordamesoderm induced by the overexpression of nodal mRNA coinjected with G-CaMP, and the left side (marked by green) was also chordamesoderm. The cells in the chordamesodermal tissues showed a relatively low frequency of calcium flashes compared with Movie S1.
(5.26 MB AVI)

Movie S3 EB3 movement in chordamesoderm treated with thapsigargin. EB3-GFP showing the direction of MT elongation in chordamesodermal cells near the boundary was more radial by the treatment of Thapsigargin. Our previous report showed that the EB3 moved toward the boundary with heterogeneous tissues.
(1.82 MB AVI)

Movie S4 Calcium dynamics of single chordamesoderm cells during attachment to the ectoderm tissue. Calcium dynamics of isolated single chordamesodermal cells (upper side; green cytoplasm and red membrane) upon their attachment to ectodermal tissue (below the red line).
(0.84 MB AVI)

Movie S5 Calcium dynamics of chordamesodermal tissue during attachment of single ectoderm cells. Calcium dynamics of the chordamesodermal tissue (lower side; green cytoplasm and red membrane) when isolated single ectodermal cells were attaching to it. The green signal was intracellular calcium indicated by Fluo 4-AM, and same result was obtained using G-CaMP.
(7.54 MB AVI)

Movie S6 Calcium activation in individual cell. Increase in the intracellular calcium in the chordamesodermal tissue by glass needle pushing. The propagation was restricted to a single cell.
(0.69 MB AVI)

Movie S7 Calcium activation in several cells as the wave. Increase in the intracellular calcium in the chordamesodermal tissue by glass needle pushing. The propagation was restricted to several cells.
(0.40 MB AVI)

Movie S8 Calcium dynamics in chordamesodermal cells underneath the silicone piece. Increase in intracellular calcium in the chordamesodermal tissue by cells crawling underneath a block of silicone.
(2.62 MB AVI)

Movie S9 High frequency of calcium flashes near the furrow of silicone block. Calcium flashes were observed near the furrow with high frequency, and the other areas of the explant show lower frequency.
(8.22 MB AVI)

Movie S10 Calcium dynamics during CE in DMZ. Live-calcium imaging of DMZ injected with G-CaMP and membrane RFP. Most of the calcium elevation was observed near the notochord-somite boundary.
(8.90 MB MOV)
