# Supplementary figures and images for: Tissue-Tissue Interaction-Triggered Calcium Elevation Is Required for Cell Polarization during Xenopus Gastrulation
Source: PLoS One. 2010 Feb 2;5(2):e8897. doi: 10.1371/journal.pone.0008897 (PMC2814847; doi:10.1371/journal.pone.0008897)

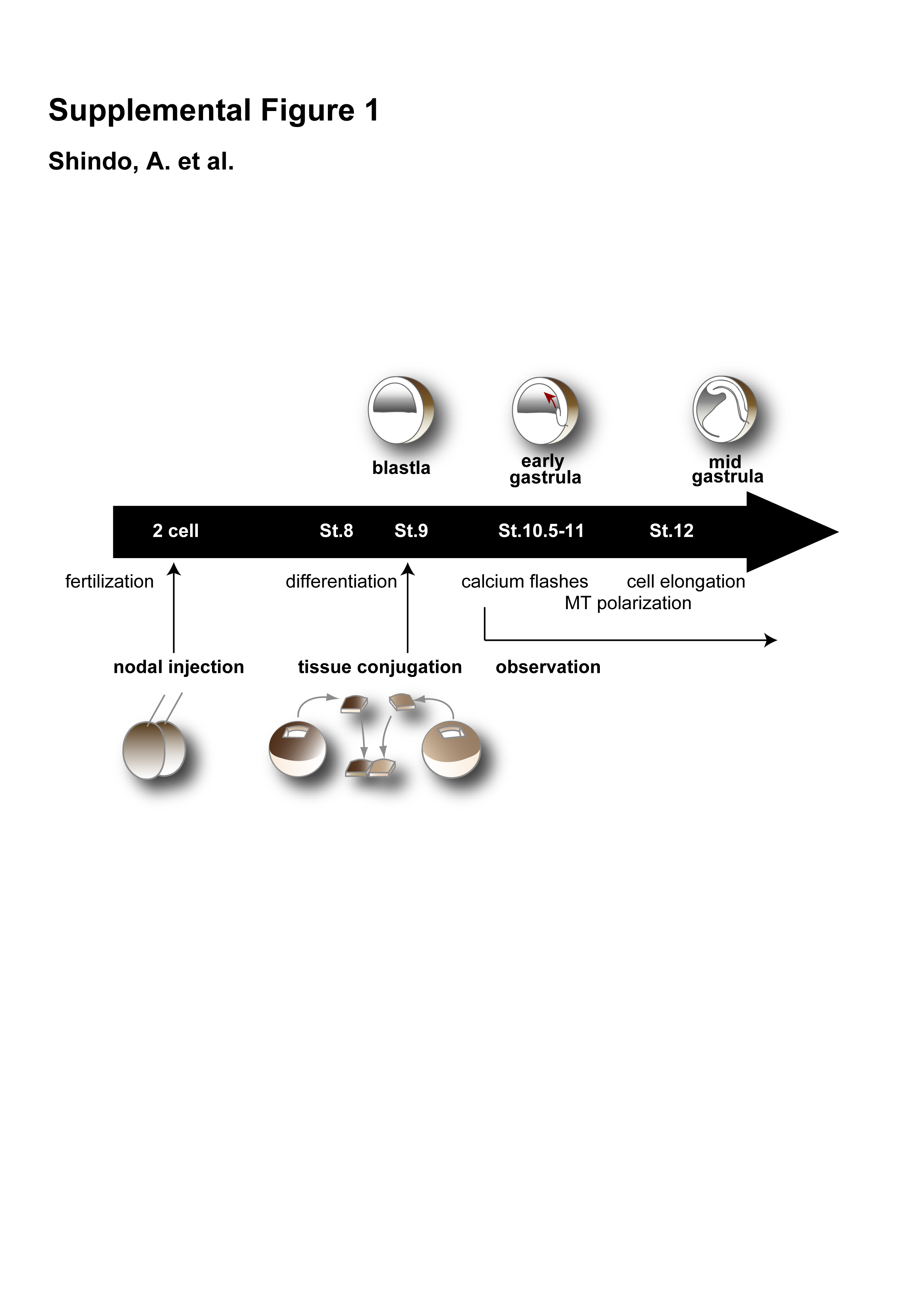

Supplement: Figure S1 — Experimental methods (time-scale). See method “Calcium imaging in the conjugation assay” (0.42 MB TIF) [file pone.0008897.s001.tif]

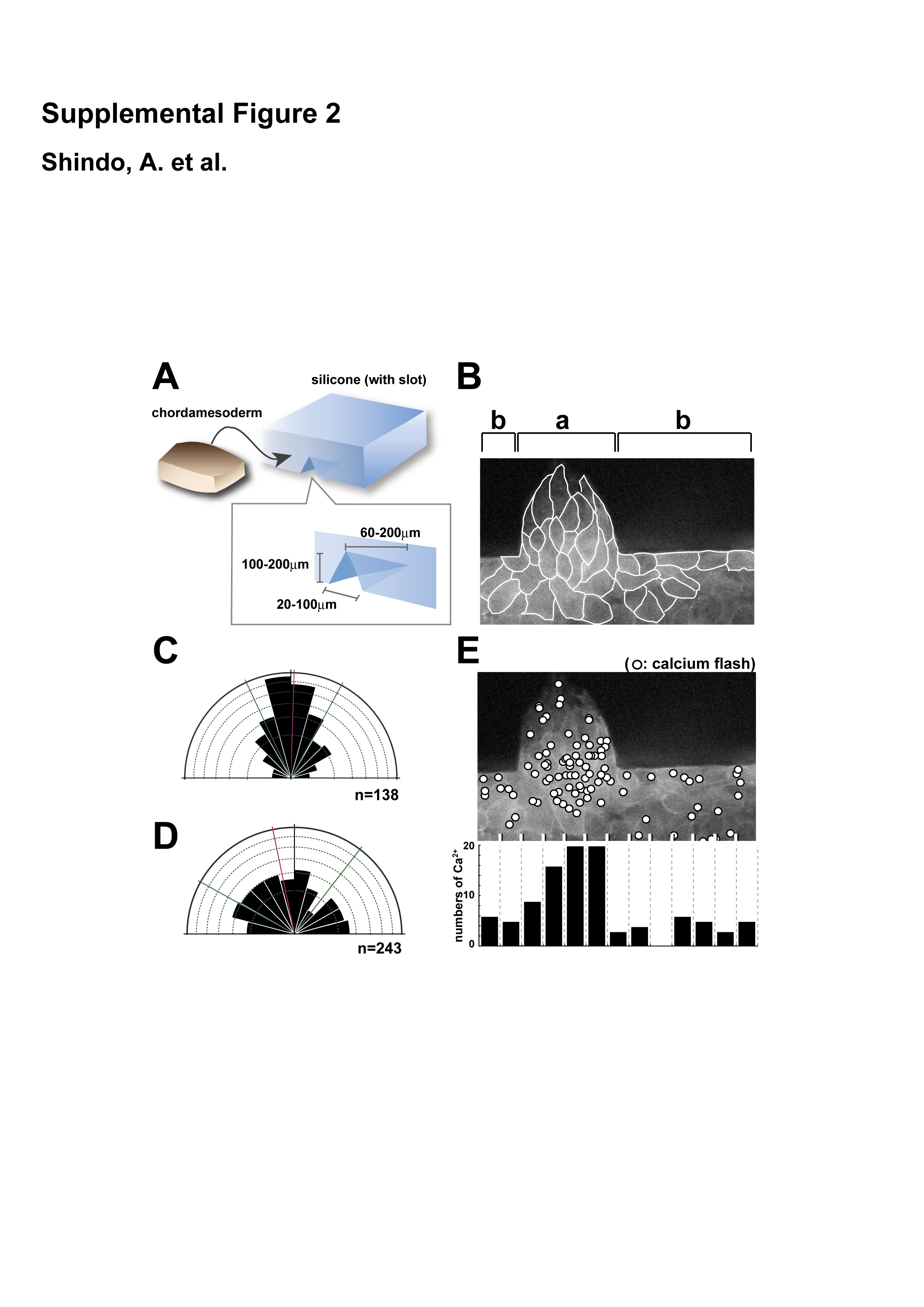

Supplement: Figure S2 — Cell alignment was altered where the apical side of the cells was underneath a silicone block. (A) The edge of a silicone block was scratched with a needle to create a furrow of the size indicated, and chordamesodermal tissue was placed beside it. (B) The cell alignment underneath the silicone furrow was coordinated (a) compared with the other area (b) (black area is the silicone block). (C) The angle of the long axis of the cells in area (B -a) was measured and is shown as a rose diagram. The underlying cells in the furrow tended to elongate and showed coordinated alignment. (D) The rose diagram showing the angle of the long axis of the cells in area (B - b). They did not show coordinated alignment. (E) The calcium flashes were observed near the furrow with high frequency (Movie S9). The dots show the sites of calcium flashes in the chordamesodermal tissue for 4 hours while cells were crawling under the silicone block (black area is the silicone block), and the number of them is indicated in the graph. (1.30 MB TIF) [file pone.0008897.s002.tif]
